# Identification of people with autosomal dominant polycystic kidney disease using routine data: a cross sectional study

**DOI:** 10.1186/1471-2369-15-182

**Published:** 2014-11-20

**Authors:** Andrew P McGovern, Simon Jones, Jeremy van Vlymen, Anand K Saggar, Richard Sandford, Simon de Lusignan

**Affiliations:** Department of Health Care Management and Policy, University of Surrey, Guildford, UK; St George’s University of London, London, UK; East Anglian Medical Genetics Service, Addenbrooke’s Hospital, Cambridge, UK

**Keywords:** Autosomal dominant polycystic kidney disease, Screening, Early detection, Primary care records

## Abstract

**Background:**

Autosomal dominant polycystic kidney disease (ADPKD) causes progressive renal damage and is a leading cause of end-stage renal failure. With emerging therapies it is important to devise a method for early detection. We aimed to identify factors from routine clinical data which can be used to distinguish people with a high likelihood of having ADPKD in a primary health care setting.

**Method:**

A cross-sectional study was undertaken using data from the Quality Intervention in Chronic Kidney Disease trial extracted from 127 primary care practices in England. The health records of 255 people with ADPKD were compared to the general population. Logistic regression was used to identify clinical features which distinguish ADPKD. These clinical features were used to stratify individual risk using a risk score tool.

**Results:**

Renal impairment, proteinuria, haematuria, a diastolic blood pressure over 90 mmHg and multiple antihypertensive medications were more common in ADPKD than the general population and were used to build a regression model (area under the receiver operating characteristic curve; 0.79). Age, gender, haemoglobin and urinary tract infections were not associated with ADPKD. A risk score (range −3 to +10) of ≥0 gave a sensitivity of 70.2% and specificity 74.9% of for detection.

**Conclusions:**

Stratification of ADPKD likelihood from routine data may be possible. This approach could be a valuable component of future screening programs although further longitudinal analyses are needed.

## Background

Autosomal dominant polycystic kidney disease (ADPKD) is a major cause of end-stage renal disease (ESRD) and may result in the need for renal replacement therapy [1]. ADPKD is the most common form of polycystic kidney disease and one of the most common genetic diseases with a reported prevalence of around 1 in 400 to 1000 and accounts for 7-10% of patients with end-stage renal disease (ESRD) [2–5]. It is genetically heterogeneous with 80-85% of cases due to mutations in *PKD1* with the remainder due to mutations in *PKD2*. Mutations in PKD2 predict a milder phenotype with ESRD occurring at a median age of 79 years compared to 58 years in individuals with a PKD1 mutation [6].

Expansion of renal cysts causes distortion and compression of normal renal tissues, intrarenal ischaemia, hyper-activation of the renin-angiotensin-aldosterone system (RAAS) and the development of sustained hypertension [7, 8]. Hypertension results in cardiovascular disease, the leading cause of death in ADPKD [9], renal function decline [10], and may also further accelerate cyst growth [11]. Treatment of hypertension with angiotensin-converting enzyme inhibitors (ACEIs) has been hypothesised to prevent or delay these effects. Longitudinal studies in people with ADPKD demonstrate ACEI use is associated with slower progression of renal disease and increased duration of survival [12, 13] although the results of the HALT study on standard and low blood pressure targets achieved using RAAS blockade on disease progression are awaited [14].

Studies using retrospective data from large registries have so far not shown any long term benefit of standard care for chronic kidney disease (CKD) on ADPKD progression [15]. However, novel therapies targeting cAMP production in the kidney have shown promise in ADPKD treatment. Trials of somatostatin analogues demonstrate a reduced rate of increase in total kidney volume but no effect on renal function has yet been identified [16–19]. A 3-year randomised controlled trial demonstrated the vasopressin receptor antagonist tolvaptan slows both cyst growth and decline in renal function compared with placebo [20] although additional safety and efficacy data have been requested before licensing [21]. Many additional drug therapies are also under investigation [22]. With potentially effective therapies now on the horizon it is important to devise a method for early detection of ADPKD in addition to cascade screening of at risk relatives within known ADPKD families.

Here we aim to identify clinical features from routinely collected data (primary care records) that could be used in the early identification of people at high risk of ADPKD. We use a cross-sectional analysis to assess whether known clinical features of ADPKD enable the condition to be distinguished from a large primary care population.

## Methods

We performed a cross-sectional analysis on people included in the Quality Improvement in Chronic Kidney Disease (QICKD) trial database. The QICKD trial was a three-arm cluster randomised controlled trial to analyses the impact of audit-based education on blood pressure control in people with renal disease [23]. The QICKD trial database comprises routinely collected data from 127 primary care practices across England. These practices are a nationally representative sample of urban, sub-urban, and rural practices in London, Surrey, Sussex, Leicester, Birmingham, and Cambridge. Anonymised patient records for all patients registered at these practices were collected in December 2010 and included all electronic patient records available up until this date.

England has a registration based primary health care system. With very few exceptions the whole population is registered with a single primary care centre. Patients access non-emergency services through their primary care practitioner who receives letters about all hospital attendances including emergencies. Since 2004 there has been a pay-for-performance system with remuneration based on extracts from routine data [24]. Primary care records in the UK therefore provide a comprehensive patient record.

The QICKD database contains primary care records extracted during the trial period (January 2006 to December 2010). In addition, historical records for each patient held at their current practice were also available. We used the data recorded between 2006 and 2010 to define the characteristics and risk factors of our population. All patients registered with their practice at the time of the final data extraction (December 2010) were included for analysis (people were excluded if they left the practice or died before the time of the final data extraction). The median time of progression to ESRD is late in the sixth decade of life [5, 10]. Our primary focus here is detection before this stage and we have therefore limited our analysis to people under 60 years old.

### ADPKD case definition

We defined ADPKD cases as anyone who had ever had one or more clinical code for polycystic kidney disease entered into their primary care record (Table 1). As many patients were not categorised by type of polycystic kidney disease and ADPKD is the most common type all these cases were included.

**Table 1:** **Read codes used to define autosomal dominant polycystic kidney disease**

Clinical code	Code term
Read codes: 5-byte version 2	
PD11.	Polycystic kidney disease
PD111	Polycystic kidneys, adult type
PD11z	Polycystic kidney disease NOS
Read codes: version 3	
PD11.	Polycystic kidney disease
PD111	ADPKD - Autosomal dominant polycystic kidney disease
X785m	Cystic kidney disease NEC
X785n	Adult type polycystic kidney disease type 1
X785o	Adult type polycystic kidney disease type II
X785p	Autosomal dominant polycystic kidney disease in childhood
XE1LN	Polycystic kidney disease NOS
XM19L	Polycystic kidneys, adult type (dominant) associated with renal failure

NOS = not otherwise specified, NEC = not elsewhere classified.

### Predictor variables

The following variables were hypothesised to be associated with identified ADPKD: Demographic variables; age and gender. Clinical variables; systolic blood pressure, diastolic blood pressure, estimated glomerular filtration rate (eGFR), serum haemoglobin, haematuria, proteinuria, the presence of urinary tract infections, and multiple antihypertensive medications. Age was defined as the patient’s age in August 2008 (the end of the first round of data extraction from the trial practices). Patient gender was available from the patient record.

The clinical variables were determined from patient records between 01 January 2006 and 31 December 2010. Several blood pressure variables were constructed to identify the method of analysing blood pressure with the strongest association with ADPKD. Systolic blood pressure was defined as the highest recorded blood pressure (recorded during the sample period; 2006 to 2010 inclusive) and the mean systolic blood pressure. Similarly diastolic blood pressure was defined as the highest diastolic blood pressure and the mean diastolic blood pressure. These variables were also converted into binary predictor variables with varying cut-offs e.g. mean systolic blood pressure over 130 mmHg.

eGFR was calculated from serum creatinine measurements using the mean of two creatinine measurements where available, if no creatinine measurement was available lab reported eGFR was used [25]. eGFR was calculated using the Modification of Diet in Renal Disease (MDRD) equation [26]. Patients were categorised by CKD stage or as renal function not measured if no creatinine or eGFR measurements were available. Serum haemoglobin was analysed as a continuous variable and as a categorical variable with four levels; polycythaemia (haemoglobin; Hb >18.0 g/dl male and >16.0 g/dl female), anaemia (Hb <13.5 g/dl male and <11.5 g/dl female), normal, and not measured. The haemoglobin value used was the earliest recorded during the sample period. Extreme values (less than 3 g/dl and more than 30 g/dl) were excluded as likely inputting errors.

Haematuria was defined as the presence of any Read code pertaining to the detection, history, or observation of blood in the urine. Proteinuria was defined using a cascading hierarchy of investigation types which we have previously described [27]. In brief, the presence of proteinuria was defined using albumin-creatinine ratio (ACR) where this was measured, if ACR was unavailable then protein-creatinine ratio (PCR) was used, then 24 urinary protein, then urinalysis (urine dipstick) results respectively. Individuals were classified as having proteinuria or no proteinuria using previously defined thresholds [27]. Where no urinary protein test was recorded the person was classified as not measured. A urinary tract infection was defined as the presence of one or more diagnostic Read codes for any urinary tract infection or laboratory confirmed identification of a pure culture of pathogenic bacteria from a urine sample.

Multiple antihypertensive medications was defined as one or more ACEI or angiotensin receptor blocker and one more calcium channel antagonist or diuretic prescribed simultaneously.

### Statistical methods

A logistic regression model was developed to identify associations between the predictor variables and the presence of ADPKD. Model selection was performed using backwards stepwise elimination of non-significant variables (p value >0.05). Odds ratios (ORs) with 95% confidence intervals were calculated for all of the variables included in the final regression model. The area under the receiver operating characteristic (AUROC) curve is reported as a measure of the predictive accuracy of the model. Additional model validation was carried out including Hosmer-Lemeshow tests for goodness-of-fit and collinearity studies. The presence of missing data was accounted for by including a data not recorded category for all relevant variables and analysing the effect of missing data as a predictor for ADPKD.

The risk model was converted into a risk stratification score. This score could be applied to primary care databases to identify people who have a high likelihood of having ADPKD and who may be suitable for a future screening program with renal ultrasound. The scores assigned to each component were calculated by multiplying the regression coefficients from the logistic regression model by two and rounding to the nearest integer. The summation of these scores across all variables gives an individual risk score for ADPKD. The sensitivity, specificity, positive predictive value (PPV), negative predictive value (NPV), and proportion of patients with ADPKD were calculated for each cut-off score.

All statistical analyses were performed using the statistical package R version 2.15.3 (The R Foundation for Statistical Computing).

### Ethical considerations

The original QICKD trial was approved by the Oxford Research Ethics Committee. Details of the ethical approval are contained in the trial registration (Current Controlled Trials reference: ISRCTN56023731. URL: http://www.controlled-trials.com/ISRCTN56023731). All data were anonymised at the point of data extraction so no patient identifiable data were used in this study. Permission for the use of the patient data collected for the trial for secondary analyses was included in the original trial ethical approval and additional permissions were sought to access the trial database. All data use was compliant with data governance requirements of the University of Surrey.

## Results

Everyone who was included in the QICKD trial database was included for analysis (n = 951,764). We excluded people who died or left their primary care practice before the final data collection in December 2010 (n = 109,701). As our focus is on early diagnosis of ADPKD we excluded all adults over 60 years old (n = 157,551). From the remaining population (N = 684,512) we found 255 people who had one or more diagnostic code for polycystic kidney disease (Table 2). This equates to a prevalence of PKD in this population of 0.04% or approximately 1 in 2700.

**Table 2:** **Read codes which had been used to record polycystic kidney disease in 255 identified cases**

Read codes: 5-byte version 2	Read codes: version 3 (equivalent)	Number of patients with code
PD11.	PD11.	202
PD111	PD111	109
PD11z	X785m/XE1LN	9

Some individuals had more than one clinical code in their medical record. Other diagnostic codes for polycystic kidney disease not included in this table were not used by clinicians or were not available in our dataset.

The mean age of people without ADPKD was 31.47 (standard error of the mean; SEM 16.88). The mean age of people with ADPKD was 41.48 (SEM 12.83). There were 331,562 (48.5%) females in the group without ADPKD and 137 (53.7%) in the group with ADPKD (Table 3). Renal impairment, proteinuria, haematuria, hypertension, urinary tract infections and use of multiple antihypertensive medications were more prevalent in people with ADPKD than people without. Missing data was more prevalent in the people without ADPKD.

**Table 3:** **The prevalence of clinical features associated with PKD in people with PKD and people without**

	People with PKD		People without PKD	
	n (%)	mean (SEM)	n (%)	mean (SEM)
Female	137 (53.7)		331,562 (48.5)	
Age	255 (100.0)	41.48 (12.83)	684,257 (100.0)	31.47 (16.88)
CKD stage				
No impairment	97 (38.0)		149,848 (21.9)	
Stage 3A	26 (10.2)		8,608 (1.3)	
Stage 3B	17 (6.7)		372 (0.1)	
Stage 4	17 (6.7)		152 (0.0)	
Stage 5	17 (6.7)		222 (0.0)	
Not measured	81 (31.8)		525,055 (76.7)	
Proteinuria				
Negative	87 (34.1)		186,446 (27.2)	
Positive	126 (49.4)		21,735 (3.2)	
Not measured	42 (16.5)		476,076 (69.6)	
Haemoglobin	29 (11.4)	14.1 g/dl (1.2)	41,243 (6.0)	13.8 g/dl (1.6)
Haematuria	31 (12.2)		22,452 (3.3)	
Urinary tract infections	26 (10.2)		24,123 (3.5)	
Mean systolic BP	27 (10.6)*	129.5 mmHg (15.9)	42,294 (6.2)	124.7 mmHg (14.8)
Mean diastolic BP	27 (10.6)*	78.7 mmHg (9.0)	42,510 (6.2)	77.1 mmHg (9.4)
Highest systolic BP	27 (10.6)*	146.2 mmHg (26.1)	42,522 (6.2)	132.4 mmHg (18.8)
Highest diastolic BP	27 (10.6)*	91.5 mmHg (16.6)	42,510 (6.2)	82.3 mmHg (11.8)
Diastolic BP over 90 mmHg	17 (6.7)		10,776 (1.6)	
Diabetes	13 (5.1)		15,988 (2.3)	
Multiple antihypertensives	53 (20.8)		8,911 (1.3)	

*Number of people with more than a single BP recording. SEM = standard error of the mean, PKD = polycystic kidney disease, BP = blood pressure.

There was a strong correlation between declining renal function and ADPKD (Table 4). Proteinuria was also more likely in ADPKD than the general population as was haematuria, and taking multiple antihypertensive medications. Diastolic blood pressure was found to have a stronger association with ADPKD than systolic blood pressure. As the two variables had a high degree of collinearity systolic blood pressure was removed from the model. Using a single cut-off value diastolic blood pressure of 90 mmHg was found to produce a binary variable with the strongest association with ADPKD (smallest p value). The following were not significantly associated with ADPKD and were therefore removed from the model; age, gender, serum haemoglobin, and the presence of urinary tract infections. The AUROC for the final model (Table 4) was 0.788.

**Table 4:** **Associations between clinical features and polycystic kidney disease in a primary care population of 684,512 people with 255 identified cases of polycystic kidney disease**

Clinical feature	Odds Ratio (95% confidence Interval)	p value	Regression score
Renal function			
No impairment detected	0.00 [reference]	-	0
Stage 3A	3.95 (2.55 - 6.12)	<0.001	3
Stage 3B	42.36 (24.20 - 74.16)	<0.001	7
Stage 4	101.27 (55.88 - 183.55)	<0.001	9
Stage 5	75.08 (42.26 - 133.39)	<0.001	9
Not measured	0.28 (0.21 - 0.38)	<0.001	−3
Proteinuria			
Negative	0.00 [reference]	-	0
Positive	1.59 (1.04 - 2.43)	0.0341	1
Not measured	0.90 (0.68 - 1.20)	0.4899	0
Haematuria present	1.85 (1.24 - 2.78)	0.0029	1
Diastolic BP over 90 mmHg	2.00 (1.20 - 3.34)	0.0078	1
Diabetes	0.31 (0.17 - 0.57)	<0.001	−2
Multiple antihypertensives	3.83 (2.66 - 5.52)	<0.001	3

BP = blood pressure.

Using the risk scores calculated from the regression model coefficients (coefficient multiplied by 2 and rounded to the nearest integer) produced a range to total individual risk scores ranging from −3 to 10 (Table 5). The possible cut-off values for total risk score produced sensitivities ranging from 70.20% to 2.75% and corresponding specificities ranging from 74.91% to 99.99%.

**Table 5:** **The predictive values of total regression score cut-offs for polycystic kidney disease**

Regression score	PPV (%)	NPV (%)	Sensitivity (%)	Specificity (%)	Detection rate
−3	0.00	100.00	100.00	0.00	1 in 2,683
−2	0.10	99.99	70.20	74.91	1 in 959
0	0.11	99.98	68.24	76.73	1 in 915
1	0.48	99.98	34.90	97.29	1 in 209
3	0.82	99.97	30.20	98.63	1 in 121
4	3.82	99.97	21.57	99.80	1 in 25
7	6.40	99.97	20.00	99.89	1 in 15
8	7.99	99.97	14.51	99.94	1 in 12
9	8.33	99.97	13.33	99.95	1 in 11
10	15.56	99.96	2.75	99.99	1 in 5

PPV = positive predictive value, NPV = negative predictive value.

## Discussion

The clinical features which best distinguished people with a diagnostic code for polycystic kidney disease from the primary care population were found to be chronic kidney disease (CKD) stage (3A or worse), proteinuria, haematuria, diastolic BP greater than 90 mmHg and being on multiple antihypertensive medications. A regression model using these features was used to create individual patient risk scores which can be used to predict the likelihood of ADPKD and could be useful as a pre-screening risk identification tool.

### Strengths and limitations

This is the first analysis that we are aware of which uses a non-specialist clinical dataset to perform an analysis of factors associated with a diagnosis of ADPKD. This has enabled the comparison of people with ADPKD with the general population and the identification of factors which could be used to identify individuals at greater risk of ADPKD from existing primary care datasets.

The prevalence 1 in 2700 of ADPKD identified here is lower than previously reported; 1 in 400 to 1000 [2–5]. However these estimates have been based on extrapolations from small samples. Our prevalence estimate is comparable to a more recent analysis that used case identification from the hospital and primary care settings; 1 in 1700 to 3000 [28]. Under-recording of ADPKD in primary care may be a contributory factor. A second factor is that ADPKD will have not yet become apparent or have been diagnosed in the younger people in the studied population. The average age of the people with diagnosed ADPKD is over 10 years higher than the average age of the people without ADPKD strongly suggesting that there is under-diagnosis in the younger population. Additional family studies to identify at risk but undiagnosed relatives of all known cases would also be important for future studies. The impact of these effects in unclear although it seems likely that the inclusion of these cases would improve the PPV of the model.

Using routinely collected data has many limitations [29]. These results are reliant on accurate recording of these data by primary care clinicians and a small amount of miscoding and misclassification has been previously noted to occur [30]. However these data are real world data which is the data available to primary care clinicians and the conclusions drawn here are likely to be generalisable and applicable in primary care.

The nature of a cross-sectional analysis is such that we were unable to determine whether abnormalities were coded into the electronic record before or after the diagnosis of ADPKD. It is likely that there is bias towards increased renal function testing, BP monitoring, and antihypertensive use following diagnosis which may be responsible for some of the associations identified here. A longitudinal analysis, using a dataset with a longer duration of follow-up, is required to establish the direction of causation in these apparent associations.

There were additional clinical features of ADPKD which we were unable to include in our model as they have not been collected from primary care records as part of the original QICKD trial data collection. These comprise; the presence of abdominal pain, renal colic, and abdominal masses. These variables may help to improve the predictive capacity of future models. Furthermore family history of ADPKD is a highly important risk factor which was not available in our dataset and should be incorporated into future models. Some of the features of included in our model such as CKD stages 3B to 5 are already indications for renal tract ultrasound scanning. This may limit the utility of this approach to case identification although other included features do not preclude investigation.

Coding of polycystic kidney disease was not specific for type of disease. As ADPKD is the most common form we have assume that all patients coded without polycystic kidney disease type are ADPKD cases. This necessarily means that our case definition for ADPKD will potentially capture a small number of other types of polycystic kidney disease.

### Comparison with the literature

To the best of our knowledge no population based screening method for ADPKD has yet been devised. Ultrasound provides a cheap and safe method for diagnosis and screening of people with high likelihood of ADPKD [31] but cost will prevent this screening from being applied to the whole population. Screening selected people with a high pre-test probability with ultrasound may be a cost effective way to identify people early on with ADPKD when effective treatment becomes available.

### Implications

These data suggest that development of a targeted ADPKD screening program is possible. This program could consist of two stages: Firstly risk stratification in primary care using routinely collected primary care data would enable the identification of high risk individuals. Secondly these people should be referred for ultrasound scanning or additional screening tests to identify or exclude ADPKD. Such a screening program is likely to prove cost effective as novel therapies for ADPKD emerge.

### Further research

Application of the models presented here to other large population datasets is needed to verify our findings. Additionally longitudinal data would help to ascertain which of the identified ADPKD predictors are most commonly measured early in the disease process. This is the next essential step in development of a tool to predict people at high risk of ADPKD and would enable refinement of the population group which should be sent for screening. Using a combination of a disease registry for ADPKD (from secondary care data) and primary care data would facilitate this aim.

## Conclusions

Whilst the limitations of the available data prevent us from conclusively demonstrating that early ADPKD case finding is possible using routine primary care data, this approach appears promising. A longitudinal analysis using linked primary and secondary care data could more conclusively demonstrate whether or not this method could be used in ADPKD screening. If this approach proves successful it could be used to form the basis for or an adjunct to an ADPKD screening program.

## Abbreviations

ACEI: Angiotensin-converting enzyme inhibitor
ACR: Albumin-creatinine ratio
ADPKD: Autosomal dominant polycystic kidney disease
AUROC: Area under the receiver operating characteristic
BP: Blood pressure
CKD: Chronic kidney disease
ESRD: End-stage renal disease
Hb: Haemoglobin
NPV: Negative predictive value
OR: Odds ratio
PCR: Protein-creatinine ratio
PKD: Polycystic kidney disease
PPV: Positive predictive value
RAAS: Renin-angiotensin-aldosterone system
QICKD: Quality Improvement in Chronic Kidney Disease [trial]
SEM: Standard error of the mean
